# Treatment Results and Prognostic Indicators in Thymic Epithelial Tumors: A Clinicopathological Analysis of 45 Patients

**Published:** 2014-07

**Authors:** Mansour Ansari, Farzin Dehsara, Mohammad Mohammadianpanah, Ahmad Mosalaei, Shapour Omidvari, Niloofar Ahmadloo

**Affiliations:** 1 Department of Radiation Oncology, Nemazee Teaching Hospital, Shiraz University of Medical Sciences, Shiraz, Iran;; 2 Student Research Committee, Nemazee Teaching Hospital, Shiraz University of Medical Sciences, Shiraz, Iran;; 3 Colorectal Research Center, Shahid Faghihi Hospital, Shiraz University of Medical Sciences, Shiraz, Iran;; 4 Shiraz Institute for Cancer Research, School of Medicine, Shiraz University of Medical Sciences, Shiraz, Iran

**Keywords:** Thymic neoplasms, Prognosis, Surgery, Radiotherapy

## Abstract

**Background:** Thymomas are rare epithelial tumors arising from thymus gland. This study aims at investigating the clinical presentation, prognostic factors and treatment outcome of forty five patients with thymoma and thymic carcinoma.

**Methods:** Forty-five patients being histologically diagnosed with thymoma or thymic carcinoma that were treated and followed-up at a tertiary academic hospital during January 1987 and December 2008 were selected for the present study. Twelve patients were solely treated with surgery, 14 with surgery followed by adjuvant radiotherapy, 12 with sequential combined treatment of surgery, radiotherapy and/or chemotherapy and 7 with non-surgical approach including radiotherapy and/or chemotherapy.  Tumors were classified based on the new World Health Organization (WHO) histological classification.

**Results:** There were 18 women and 27 men with a median age of 43 years. Twelve patients (26.7%) had stage I, 7 (17.8%) had stage II, 23 (51%) had stage III and 2 (4.5%) had stage IV disease. Tumors types were categorized as type A (n=4), type AB (n=10), type B1 (n=9), type B2 (n=10), type B3 (n=5) and type C (n=7). In univariate analysis for overall survival, disease stage (P=0.001), tumor size (P=0.017) and the extent of surgical resection (P<0.001) were prognostic factors. Regarding the multivariate analysis, only the extent of the surgical resection (P<0.001) was the independent prognostic factor and non-surgical treatment had a negative influence on the survival. The 5-year and 10-year overall survival rates were 70.8% and 62.9%, respectively.

**Conclusion:** Complete surgical resection is the most important prognostic factor in patients with thymic epithelial tumors.

## Introduction

Thymomas are rare epithelial tumors arising from thymus gland and accounts for 30% of all primary anterior mediastinal masses in adults. They are mainly common among men and their occurrence increases in middle age reaching its peak in the seventh decade of life.^1^^,^^2^ Thymomas are usually encapsulated very slow growing and locally invasive tumors. Thymic cancers usually disseminate to the pericardium, pleura or diaphragm; and rarely metastasize to distant extrathoracic sites such as bone or liver. These neoplasms are well known to have a propensity for late relapse even following a complete resection.^2^^,^^3^ Most thymomas are often asymptomatic at diagnosis and discovered incidentally during an elective chest radiograph; however, they may have various presentations. Prognosis depends on the stage of the disease, tumor resectability, histological tumor pattern, tumors behavior and patient’s performance status.^2^ Complete surgical resection is considered as the treatment of choice for thymoma. Although chemotherapy and/or radiotherapy have been suggested for locally advanced and metastatic stages, the efficacy of these treatment modalities remains controversial.^2^^,^^4^^,^^5^ Complete surgical resection is highly curative and offers the best chance for long-term survival in early-stage thymomas, however survival rates decline in locally advanced and metastatic diseases. Therefore, early detection and prompt surgical intervention is highly suggested.^2^^,^^3^ There is no data regarding the World Health Organization (WHO) histologic classification of thymoma in Iran. A few articles that are available refer to case reports or related to myasthenia gravis and not thymoma. To the best of our knowledge, this is the first study in this area. The aim of this study was to investigate the clinical presentation, prognostic factors and treatment outcome of 45 patients with thymic epithelial tumors.

## Materials and Methods

This retrospective study was carried out at the radiation oncology department of Nemazee Hospital. The characteristics, prognostic factors and survival of all patients with histologically proven thymoma or thymic carcinoma (treated and followed-up between January 1987 and December 2008) were analyzed. In this study, the Masaoka staging system of thymoma was used for the classification of thymic neoplasms. According to this staging system, tumors that were completely encapsulated and lacked microscopic capsular invasion were considered as stage I. Tumors with microscopic capsular invasion or macroscopic invasion into mediastinal pleura or surrounding fatty tissue were classified as stage II; whereas stage III included the tumors with macroscopic invasion into adjacent organs such as great vessels, lung or pericardium. Finally, stage IV, were tumors with pleural or pericardial dissemination (IVa), hematogenous or lymphogenous distant metastasis (IVb).^6^ The histologic subtype of thymoma was defined according to the new WHO histologic classification.^7^ Post treatment follow-up for patients included a history and physical examination, with chest X-ray performed at the outpatient clinic every 3 months for the first 2 years, every 4 months during the third year, every 6 months in the fourth and fifth years and annually thereafter. Chest CT scan was checked annually.

*Surgery*

Surgical technique included a median sternotomy approach in all cases. Total thymectomy with or without en bloc removal of adjacent mediastinal fat and pleura was performed in 33 patients.

*Radiotherapy*

External beam radiotherapy megavoltage machines (telecobalt units or linear accelerator) were used and a median total dose of 45 (ranged 30-60) Gy was delivered. Two dimensional (2D) radiation techniques included anterior wedge-pair portals or anterior-posterior opposed fields with or without anteriorly weighted. All treatments were delivered using conventional fractionation as daily fraction of 1.8-2 Gy and five fractions per week. 

*Chemotherapy*

Chemotherapy consisted of cyclophosphamide, doxorubicin and cisplatin with or without prednisolone (CAP regimen) in 13 patients and cyclophosphamide, vincristine, prednisolone and cisplatin in 4 patients. 

*Definition of Survival*

Date of surgery or core needle biopsy was considered as the time of diagnosis. The survival durations were calculated from the date of surgery or core needle biopsy until the events of tumor regrowth, death due to any cause or the last follow-up. Disease free survival was defined from the date of diagnosis to the date of disease recurrence at any sites. Overall survival was defined from the date of diagnosis to date of death due to any cause. 

*Statistics*

Clinical and pathological variables were analyzed using SPSS for Windows version 17.0 statistical software (SPSS, Chicago, IL). Univariate analysis for disease free survival and overall survival rates were performed using the Kaplan–Meier method and prognostic factors were compared using the log-rank test. Multiple-covariate analysis was performed using the stepwise regression hazards regression model. The hazard ratio (HR) for death, with the 95% confidence interval (CI), was calculated for the variable groups. The stratified log-rank test was used to compare treatment results in each variable group. A P value of 0.05 or less was considered to be statistically significant. 

## Results

There were 18 women and 27 men ranging from 17 to 78 years, with a median and mean age of 43 and 45.4±17.7 years respectively at diagnosis. The peak incidence was during the fifth decade of life in both genders. Twenty-five patients were less than or equal to 45 years old at presentation and 20 patients were more than 45 years old. Cough (60%), dyspnea (51%), chest pain (29%), weakness (24%), and ptosis (20%) were the most frequent presentation followed by dysphagia (7%), Cushing’s syndrome (4%) and SVC syndrome (4%). In addition, the signs and the symptoms of myasthenia gravis were detected in 11 patients (24.5%). Twelve patients (26.7%) had stage I, 7 (17.8%) had stage II, 23 (51%) had stage III and 2 (4.5%) had stage IV disease (table 1). The median largest tumor diameter was 7 cm (range 4-15). The distribution of histologic subtype was type A (n=4), type AB (n=10), type B1 (n=9), type B2 (n=10), type B3 (n=5) and type C (n=7) as shown in table 2. No association between histologic subtypes and age, sex, stage of disease, tumor size, and type of surgery was observed.

**Table 1:** Distribution of disease stage and treatment schedule in 45 patients with thymic epithelial neoplasms

**Stage of disease**	**Treatment schedules**					**Total**
	**S+RT**	**S+RT+CT**	**S+CT**	**S**	**NST**	
Stage I	0	0	0	12	0	12
Stage II	7	1	0	0	0	8
Stage III	7	9	1	0	6	23
Stage IV	0	0	1	0	1	2
Total	14	10	2	12	7	45

S: Surgery; RT: Radiotherapy; CT: Chemotherapy; NST: Non Surgical Treatments (Radiotherapy and/or chemotherapy)

**Table 2:** Distribution of disease stage and WHO histologic type in 45 patients with thymic epithelial neoplasms

**WHO histologic types**	**Stage of disease**				**Total**
	**Stage I**	**Stage II**	**Stage III**	**Stage IV**	
Type A	1	3	0	0	4
Type B1	1	1	7	0	9
Type B2	5	1	4	0	10
Type B3	0	1	3	1	5
Type AB	4	1	5	0	10
Type C	1	1	4	1	7
Total	12	8	23	2	45

*Radiotherapy and/or chemotherapy

Twelve patients were solely treated with surgery, 14 with surgery followed by adjuvant radiotherapy, 12 with sequential combined treatment of surgery, radiotherapy and/or chemotherapy and 7 with non-surgical approach including radiotherapy and/or chemotherapy (table 1). Total gross resection (with or without microscopic residual disease) was performed in 33 patients (73.3%), subtotal resection or debulking surgery (with gross residual disease) in 3 patients (6.7%) and core needle biopsy in 9 patients (20%) as listed in table 3. Thirty patients received external beam radiotherapy, 6 patients treated with radiotherapy alone or concurrent chemoradiation and 24 patients received postoperative radiotherapy. Patients with stage IV disease were treated with short course radiotherapy (20-30 Gy in 1-2 weeks); whereas in stage II-III, a total dose of 45-50 Gy for microscopic residual disease and 50-60 Gy for gross residual disease was delivered. Seventeen patients with stage II-IV disease received a median 4 (range 2-6) cycles of chemotherapy. As presented in table 4, patients received chemotherapy in induction setting (6 cases), adjuvant setting (3 cases) or both (8 cases).

**Table 3:** Distribution of disease stage and type of surgery in 45 patients with thymic epithelial neoplasms

**Stage of disease**	**Type of surgery**			**Total**
	**Only biopsy**	**PR**	**TGR**	
Stage I	0	0	12	12
Stage II	0	0	8	8
Stage III	7	3	13	23
Stage IV	2	0	0	2
Total	9	3	33	45

PR: Partial resection; TGR: Total gross resection

**Table 4:** Distribution of treatment schedule and WHO histologic type in 45 patients with thymic epithelial neoplasms

**WHO histologic types**	**Treatment schedules**					**Total**
	**S+RT**	**S+RT+CT**	**S+CT**	**S**	**NST**	
A	3	0	0	0	1	4
B1	5	1	2	0	1	9
B2	2	1	1	1	5	10
B3	1	2	1	1	0	5
AB	3	3	0	0	4	10
C	0	3	3	0	1	7
Total	14	10	7	2	12	45

S: Surgery; RT: Radiotherapy; CT: Chemotherapy; NST: Non surgical treatments (Radiotherapy and/or chemotherapy)

**Figure 1:**
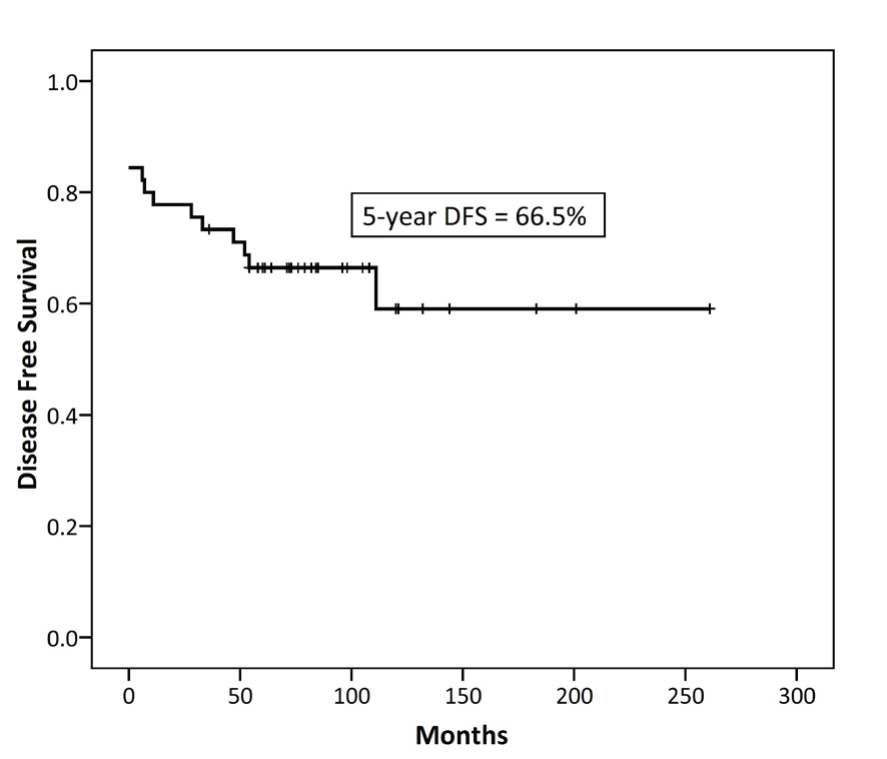
Kaplan–Meier survival curves of 5-year disease-free survival in 45 patients with thymic epithelial neoplasms.

**Figure 2:**
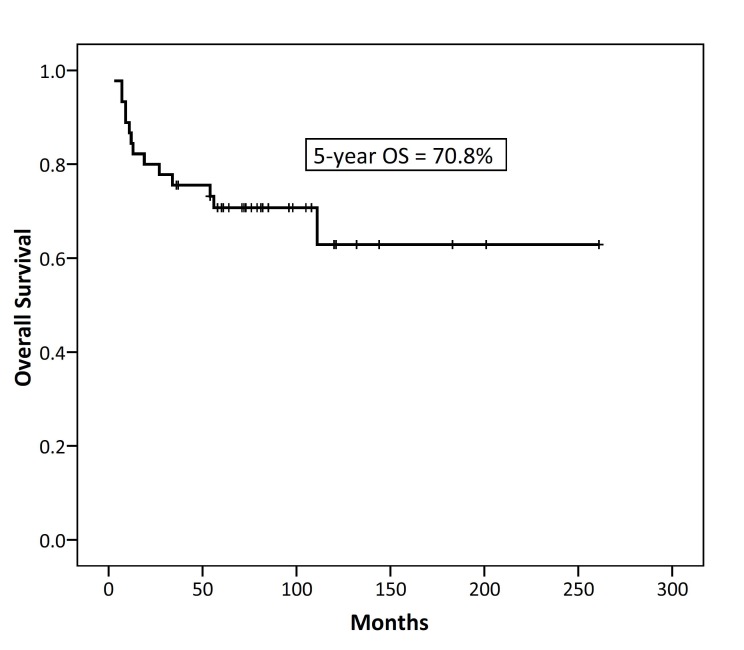
Kaplan–Meier survival curves of 5-year overall survival in 45 patients with thymic epithelial neoplasms.

After a median follow-up of 82 (range 36-261) months for surviving patients, 29 patients (64.5%) were alive and without disease, 2 (4.5%) were alive with disease, and 14 (31%) died due to the disease. Six patients developed local recurrence, 2 developed distant failure and 8 patients developed local and distant failure. The 5-year and 10-year disease free survival rates were 66.5% and 59.1% respectively; as shown in figure 1. The 5- and 10-year overall survival rates were 70.8% and 62.9% respectively; as shown in figure 2.

**Figure 3:**
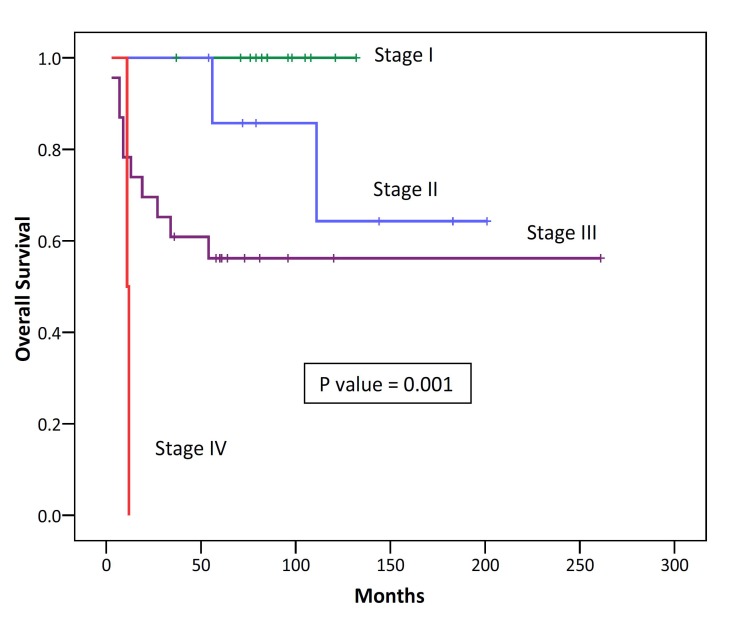
Kaplan–Meier survival analysis of overall survival categorized according to the stage of disease in 45 patients with thymic epithelial neoplasms.

**Figure 4:**
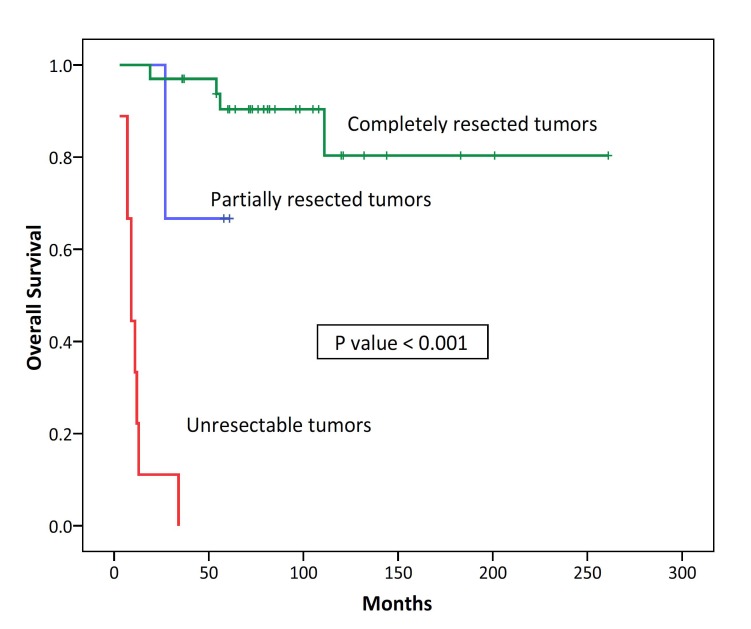
Kaplan–Meier survival analysis of overall survival categorized according to the extent of surgical resection in 45 patients with thymic epithelial neoplasms.

In univariate analysis for disease free survival, disease stage (P=0.001) and the extent of surgical resection (P<0.001) were prognostic factors. Sex, age, myasthenia gravis association, tumor size and the WHO histologic subtype were not prognostic factors for DFS (table 5). In univariate analysis for overall survival, disease stage (P=0.001), tumor size (P=0.017) and the extent of surgical resection (P<0.001) were prognostic factors (figures 3 and 4). Sex, age, myasthenia gravis association and the WHO histologic subtype were not be prognostic factors for OS (table 6).

**Table 5:** Univariate analysis of prognostic factors for overall survival in 45 patients with thymic epithelial neoplasms

**Prognostic factors**	**Patients No**	**5-year DFS rate (%)**	**P value**	**Hazard ratio (HR)**	**95% CI**
Age					
≤45 years	25	68.0	0.556	1.332	0.496-3.572
>45 years	20	64.3			
Sex					
Male	27	59.3	0.341	1.639	0.569-4.723
Female	18	77.4			
Stage of disease					
I	12	91.7	0.001	3.300	1.443 -7.550
II	8	87.5			
III	23	51.5			
IV	2	00.0			
Myasthenia gravis association					
No	34	58.4	0.043	5.990	0.790-45.436
Yes	11	90.9			
Tumor size					
<8 cm	26	76.9	0.071	2.391	0.873-6.551
≥8 cm	19	52.1			
Extent of surgical resection					
Total gross resection	33	87.7	<0.001	19.272	5.689-65.287
Non total resection	12	8.3			
WHO histologic subtype					
A	4	75.0	0.140	1.195	0.870-1.642
AB	10	80.0			
B1	9	77.8			
B2	10	68.6			
B3	5	20.0			
C	7	42.9			

OS: Overall survival; CI: Confidence interval; WHO: World health organization

**Table 6:** Univariate analysis of prognostic factors for overall survival in 45 patients with thymic epithelial neoplasms

**Prognostic factors**	**Patients’ No**	**5-year DFS rate (%)**	**P value**	**Hazard ratio (HR)**	**95% CI**
Age					
≤45 years	25	71.8	0.574	1.350	0.470-3.881
>45 years	20	69.2			
Sex					
Male	27	63.0	0.263	1.912	0.599-6.101
Female	18	82.5			
Stage of disease					
I	12	100.0	0.001	4.598	1.728 -12.234
II	8	85.7			
III	23	56.2			
IV	2	00.0			
Myasthenia gravis association					
No	34	61.3	0.017	34.522	0.257-4637.288
Yes	11	100.0			
Tumor size					
<8 cm	26	84.4	0.019	3.519	1.150-10.765
≥8 cm	19	51.5			
Extent of surgical resection					
Total gross resection	33	90.4	<0.001	21.044	5.574-79.440
Non total resection	12	16.7			
WHO histologic subtype					
A	4	75.0	0.038	1.272	0.896-1.805
AB	10	80.0			
B1	9	77.8			
B2	10	68.6			
B3	5	20.0			
C	7	42.9			

OS: Overall survival; CI: Confidence interval; WHO: World health organization

On multivariate analysis, only non-surgical treatment [Hazard ratio (HR)=0.112, 95% confidence interval (CI)=0.038-0.328, P<0.001] was the independent prognostic factor and had a negative influence on the overall survival. 

## Discussion

Thymomas usually occur in the sixth decade of life. In the present study, the median age of the patients was 42 years, which was a decade younger than that of the reported series in the literature.^8^^-^^11^ In most reported series in the literature there was no significant sex predilection.^8^^,^^10^^-^^14^ Male/female ratio in this report was 1.5 which was within the range of the reported series. In the literature, the rate of complete surgical tumor resection ranges from 63% to 100% in patients with stage I-IV thymoma.^10^^,^^13^^,^^14^ In the current study, the rate of complete tumor resection was 79% which was consistent with the results of the literature.

A surgically oriented staging system developed by Masaoka is widely used for staging of thymic epithelial tumors.^7^ According to this staging system, the distributions of the patients’ stage in this study were; stage I at 26.7%, stage II at 17.8%, stage III at 51% and stage IV at 4.5%. Such distribution is consistent with the studies of Ariyasu et al., Chen et al. and Su et al.^5^^,^^12^^,^^15^ However, it is inconsistent with the reports of Rena et al., Lucchi et al. and Ruffini et al.^11^^,^^16^^,^^17^

Currently, the WHO classification is the most common and predictive histologic classification used in thymic epithelial tumors.^7^^,^^18^ According to this classification, histologic types of thymic epithelial neoplasms are type A, AB, B1, B2, B3 and C. The distribution of histologic subtype in a study performed by Chen et al. were namely; type A (4%), type AB (34%), type B1 (8.5%), type B2 (19.5%), type B3 (13.5%), type C (18%)and unclassified tumors (2.5%).^18^ In the current study, this distribution was type A (9%), type AB (22%), type B1 (20%), type B2 (22%), type B3 (11%) and type C (16%) which is relatively similar to those of Jiao et al., Kim et al. and Kondo et al.^19^^-^^21^ Overall, 2% to 10% of all thymic epithelial neoplasms cannot be classified according to the WHO classification and are defined as combined or unclassified thymomas.^18^^,^^22^

In the series of this study, the 5-year and 10-year DFS rates were 66.5% and 59.1% respectively. This 5-year DFS survival rate is relatively consistent with the study of Huang et al. in which the 5-year DFS survival rate of 97 patients was 75%. However, DFS rates in the current study were generally lower than the most reported studies in the literature.^23^^,^^24^ In addition, in the present study, the 5-year and 10-year overall survival rates were 70.8% and 62.9% respectively which were consistent with the results of the reports by Gadalla et al and Patel et al.^8^^,^^10^ However, it was lower than those of Chan et al., Sakamoto et al. and Nakagawa et al.^9^^,^^23^^,^^24^ Lower disease-free survival in the series of the present study may be due to the lower rate of patients with stage I disease and higher rate of patients with thymic carcinoma.

Surgery is the mainstay of successful treatment and a cure for early stage thymomas; and unresectable tumors tend to have poor outcome.^2^^,^^25^ Surgical resectability and disease stage are the most important and independent prognostic factors for overall survival.^2^^-^^4^^,^^13^^,^^15^ In the present study, complete surgical resection was the most important prognostic factor for overall survival.

Few reports considered the prognostic impact of the primary tumor size in local control or survival;^9^ however, other reports were not in agreement with this conclusion.^5^^,^^19^^,^^23^^,^^26^ In addition, the prognostic impact of myasthenia gravis on the survival of patients with thymoma is controversial. Some studies found myasthenia gravis as a prognostic factor for survival in patients with thymoma; however, in most of these studies myasthenia gravis was associated with disease stage and histologic types but not with an independent prognostic factor.^5,12,13,17,18,24^ On the contrary, other reports did not find such an association.^19^^,^^23^^,^^27^^-^^30^ In this study, myasthenia gravis was not a prognostic factor but rather primary tumor size was a prognostic factor for overall survival.

Similar to the approach in the present study, patient variables including age and sex were analyzed in other studies but were not prognostic factor.^19^^,^^29^^-^^32^ In most reported studies in the literature, the WHO histologic classification was found to be an independent prognostic factor for the survival or correlated with the disease stage or tumor behavior.^12,13,16,17,24,31^ In the series of the present study, the WHO histologic classification was not found to be a prognostic factor or any association between it and other prognostic factors. This could be due to the small sample size used in each subgroup.

Adjuvant radiotherapy plays an important role in the treatment of invasive and incompletely excised thymomas.^2^^,^^4^^,^^25^ There are many reports that indicate adjuvant radiotherapy improves disease-free and/or overall survival in stage II and/or III thymomas. Subsequently, despite the lack of prospective clinical trials, adjuvant radiotherapy is recommended for all stage II and III thymomas.^5,10,18,23,25,33,34^ The use of the new radiotherapy techniques including intensity-modulated radiation therapy (IMRT) or three-dimensional computed tomography (CT)-based plan using high megavoltage photonscreated by linear accelerators, is highly recommended for minimizing the total radiation dose delivered to the critical surrounding mediastinal organs including the heart and the spinal cord.^35^ Chemotherapy is considered as a useful treatment option in patients with unresectable or metastatic thymomas.^2^^,^^4^^,^^25^ Induction chemotherapy and/or chemoradiation may improve progression-free and/or overall survival in patients with locally advanced and metastatic diseases. Consequently, induction or adjuvant chemotherapy is recommended for unresectable and stage III disease with postoperative gross residual disease or thymic carcinoma subtype.^5^^,^^18^^,^^31^^,^^33^^,^^34^ Therefore, in the present study, adjuvant radiotherapy was considered for all stage II thymomas. Patients with stage III disease received combined adjuvant radiotherapy and chemotherapy; and in unresectable stage III disease, radiotherapy or chemotherapy alone or combined chemoradiation was delivered. It is found that combined treatments are associated with a better overall survival. However, due to the small number of patients in each treatment groups, it was not possible to show the impact of radiotherapy and chemotherapy on survival.

## Conclusion

The result of this study indicates that complete surgical resection and disease stage are the most important independent prognostic factors for treatment outcome in patients with thymoma and thymic carcinoma. Advanced stage disease and unresectable tumors predict worse survival and poor outcome. 
